# Personal

**Published:** 1898-06

**Authors:** 


### PERSONAL.

---

DR. FRANCIS T. METCALFE, of Buffalo, has been appointed acting assistant-surgeon for duty on the hospital ship John Englis, U. S. Army. Dr. Metcalfe has recently returned from a European tour and on his arrival at New York found telegraphic orders directing him to report at once.

---

THE following removals and changes of address are announced :

Dr. Joseph Spangenthal, from 108 E. Genesee to 629 Main street.

Dr. H. Mickle, from 26 Linwood to 526 Delaware avenue.

Dr. A. W. Bayliss, from 378 Genesee to 592 Spring street.

Dr. A. B. Wilson, from 78 Fargo avenue to 51 Eastwood place.

---

DR. DANIEL LEWIS, of New York city, was reëlected as president of the state board of health at its annual meeting held at Albany, May

20, 1898. Dr. Smith, of Syracuse, and Dr. Jones, of Rochester, were selected as a committee to take charge of all questions relating to tuberculosis which may come before the board.

---

Dr. N. R. Coleman, of Columbus, has been elected president of the Ohio state medical society.

